# Circulating Omentin-1 as a Biomarker at the Intersection of Postmenopausal Breast Cancer Occurrence and Cardiometabolic Risk: An Observational Cross-Sectional Study

**DOI:** 10.3390/biom11111609

**Published:** 2021-10-30

**Authors:** Gerasimos Socrates Christodoulatos, Georgios Antonakos, Irene Karampela, Sotiria Psallida, Theodora Stratigou, Natalia Vallianou, Antigoni Lekka, Ioanna Marinou, Evaggelos Vogiatzakis, Styliani Kokoris, Athanasios G. Papavassiliou, Maria Dalamaga

**Affiliations:** 1 Department of Biological Chemistry, Medical School, National and Kapodistrian University of Athens, 75 Mikras Asias, Goudi, 11527 Athens, Greece; gerchristod82@hotmail.com (G.S.C.); psallidasotiria@gmail.com (S.P.); papavas@med.uoa.gr (A.G.P.); 2 Laboratory of Clinical Biochemistry & Laboratory of Hematology and Blood Bank Unit, Medical School, National and Kapodistrian University of Athens, Attikon General University Hospital, 1 Rimini Street, Chaidari, 12462 Athens, Greece; georgiosantonakos@yahoo.gr (G.A.); skokori@med.uoa.gr (S.K.); 3 2nd Department of Critical Care, Medical School, University of Athens, Attikon General University Hospital, 1 Rimini Street, Chaidari, 12462 Athens, Greece; eikaras1@gmail.com; 4 Department of Internal Medicine and Endocrinology, Evaggelismos General Hospital, 45-47 Ypsilantou Street, 10676 Athens, Greece; theodorastratigou@yahoo.gr (T.S.); natalia.vallianou@hotmail.com (N.V.); 5 Laboratory Department, NIMTS-Army Share Fund General Hospital, 12 Monis Petraki and Vasilissis Sofias Avenue, 11528 Athens, Greece; antilekka@hotmail.com; 6 Laboratory Department, Sotiria Athens General Hospital, 152 Mesogeion Avenue, 11527 Athens, Greece; ioannachond@yahoo.gr (I.M.); vogia2@gmail.com (E.V.)

**Keywords:** adipokine, breast cancer, cancer, cardiometabolic risk, cardiovascular disease, intelectin, Mediterranean diet, obesity, omentin, postmenopausal

## Abstract

Aberrant circulating omentin-1, which is an anti-inflammatory and pro-apoptotic adipokine, has been reported in various solid tumors. Therefore, we investigated whether circulating omentin-1 could be associated with postmenopausal BC (PBC) and could be used as a potential diagnostic and clinical tool taking into consideration clinicopathologic features, tumor markers, as well as anthropometric, metabolic, and inflammatory parameters. Serum omentin-1, tumor markers (CA15-3 and CEA); metabolic (insulin, glucose, HOMA index, and serum lipids), anthropometric (BMI, waist circumference, and fat mass), and inflammatory (TNF-α, IL-6, hsCRP) parameters; classic adipokines (leptin and adiponectin); the Mediterranean diet (MedDiet) score; and cardiovascular (CVD) risk were determined in 103 postmenopausal women with pathologically confirmed incident invasive BC, 103 controls matched on age, 51 patients with benign breast lesions (BBL), and 50 obese postmenopausal women of similar age. The mean serum omentin-1 was significantly lower in cases than in controls and patients with BBL (*p* < 0.001). In the patients, omentin-1 was inversely associated with tumor, metabolic and inflammatory biomarkers, cancer stage, and the number of infiltrated lymph nodes (*p* < 0.05). In all study participants, omentin-1 was negatively correlated with CVD risk and positively correlated with MedDiet score. Lower circulating omentin-1 was independently associated with PBC occurrence above and beyond known risk factors. According to the ROC curve analysis, the overall diagnostic performance of omentin-1 (0.84, 95% CI 0.79–0.89) is similar to CA15-3. Circulating omentin-1 may be a biomarker at the intersection of PBC and cardiometabolic risk in postmenopausal women, and could be modulated by the adoption of a MedDiet. Further mechanistic and large multicentric prospective and longitudinal studies are required to elucidate the ontological role of omentin-1 in BC and CVD risks, as well as its diagnostic and prognostic ability and its therapeutic potential.

## 1. Introduction

Breast cancer (BC) constitutes the most frequent type of malignancy amid women, contributing significantly to the global health burden with an estimated 2.3 million new cases globally [1,2,3,4]. Besides the well-established risk factors including age, genetic susceptibility, reproductive and hormonal factors, lifestyle and environmental determinants (sedentary life, alcohol consumption, diet, etc.), obesity, and metabolic syndrome (Mets) have also been associated with an increased risk of BC, particularly, postmenopausal BC (PBC) [5,6,7,8,9,10].

Indeed, BC, obesity, and Mets follow a parallel increasing trend globally, attributed mainly to an energy-dense diet and physical inactivity [8,9,10]. Excess body weight may be responsible for at least 7% of all PBC [1]. The overgrowth and dysfunctionality of the adipose tissue in obesity, particularly, visceral obesity, may lead to substantial alterations in its cellular composition, as well as in the dysregulation of adipocytokines that synergically promote chronic subclinical inflammation, insulin resistance, microenvironmental and cellular perturbations, and tumor transformation [4,7,11,12]. While classic adipocytokines, such as leptin and adiponectin, have been associated with BC in in vitro, animal and epidemiologic studies, little is known about novel adipocytokines [7,10,13,14].

Omentin, also known as intestinal lactoferrin receptor ITLN1 or intelectin-1, represents a novel hydrophilic 34 kDa adipocytokine of 313 amino acids, which is expressed mostly by visceral fat and exhibit pleiotropic inflammatory, metabolic, and immune-related actions [15,16]. Omentin-1 is the predominant abundant circulating isoform in the plasma, presenting 83% amino acid identity with omentin-2 and it is encoded by a gene in the 1q21.3 chromosomal region, which is associated with diabetes mellitus type 2 (T2DM) in many subjects [17,18,19]. Paradoxically, although omentin-1 is mainly secreted by the visceral adipose tissue, circulating levels of omentin-1 are downregulated in obesity-associated metabolic disorders, i.e., insulin resistance and T2DM, following the pattern of adiponectin [14,20]. Interestingly, the bulk of evidence has shown inverse associations between circulating omentin-1 and obesity, Mets, DM, polycystic ovary syndrome, cardiovascular disease (CVD), and endothelial dysfunction [18,19,21], while aberrant serum omentin-1 with either increased or decreased levels has been reported in solid malignancies [14,22,23].

The identification of novel diagnostic, prognostic, and predictive biomarkers as non-invasive tools and/or therapeutic targets in BC is currently a hot research topic. Since dysregulated circulating omentin-1, an anti-inflammatory and pro-apoptotic adipokine has been reported in various solid tumors and obesity-associated metabolic disorders, we hypothesize that postmenopausal women with BC, an obesity-related cancer, may present aberrant circulating levels of omentin-1 that could be associated with cardiometabolic risk factors, such as metabolic, lipid, and inflammatory biomarkers. To date, only four small case-control studies have examined the association between serum omentin and BC [24,25,26,27] without exploring its association with clinicopathologic, cardiometabolic, inflammatory, and dietary biomarkers. Therefore, our main goal was to evaluate the association of serum omentin-1 with PBC in the context of a cross-sectional study, taking into consideration several clinicopathologic characteristics including serum tumor markers, as well as anthropometric, metabolic, dietary, and inflammatory variables in postmenopausal women with BC, benign breast neoplasms (BBL), and obesity, and in healthy control participants. Secondary aims were to study: (1) the relationship between omentin-1 with anthropometric, cardiometabolic, dietary, and inflammatory parameters, as well as CVD risk assessed by the Framingham risk score algorithm; (2) the independent predictors of circulating omentin-1 levels in study participants; and (3) the potential diagnostic and clinical utility of serum omentin-1 in postmenopausal women with BC.

## 2. Materials and Methods

### 2.1. Study Protocol and Population

This was a cross-sectional study which took place at the Veterans’ Administration General Hospital of Athens (NIMTS) and the Attikon General University Hospital, Athens, Greece from October 2003 to January 2015 inclusive. The study population comprised 307 Greek postmenopausal women (103 patients with PBC, 103 healthy participants, 51 patients with benign breast lesions (BBL), and 50 subjects with obesity defined as a body mass index (BMI) ≥30 kg/m^2^) younger than 85 years. All participants were of Greek nationality and the same residency (Attica and Metropolitan area of Athens, Athens, Greece). We included participants with no missing data.

Eligible cases consisted of consecutive postmenopausal women with incident, pathologically confirmed, invasive ductal BC from the Surgery and Internal Medicine Departments, under age 85 (median age 59, range 50–85 years), who had not received any type of therapy such as surgery, chemotherapy, and/or radiotherapy before sample collection.

The postmenopausal healthy control group (median age 61, range 49–85 years), the group of patients with BBL (median age: 60, range 50–85 years), and the group of subjects with obesity (median age 61, range 49–84 years) were randomly selected from the outpatient clinics of the internal medicine and the laboratory departments of the same hospitals among women who were submitted to an annual check-up examination and presented a negative mammogram result indicating absence of BC. These participants were included if they had never been diagnosed with any form of cancer (except non-melanoma skin cancer). In particular, for every eligible case, an attempt was made to randomly identify a healthy control participant from the outpatient department matched to cases on age (±5 years) and as closely as possible in time to the diagnosis and sampling of the corresponding case (±1 month). Moreover, based upon the mammogram and cytopathology reports, a group of 51 women with BBL and a group of women with obesity of similar age were also selected in order to evaluate the possible effects of BBL and obesity on omentin-1.

All study participants did not present any infection at the time of enrolment in the study. Other exclusion criteria included endocrine disorders, DM, CVD, renal disease, malignancy, liver disease, autoimmune disorder, muscular, neurologic, or psychiatric disorders and routine intake of any drugs including drugs for hyperlipidemia and thyroid disorders.

Women were considered postmenopausal at the time of blood collection if they had no menstrual cycles in the last 12 months or reported a bilateral oophorectomy. Postmenopausal women with follicle-stimulating hormone (FSH) above 40 IU/L were included in the study. The mean time since menopause was similar across groups. All study participants provided a written informed consent. The study protocol was in accordance with the Declaration of Helsinki of 1964 and successive revisions, and was approved by the Institutional Review Board of both hospitals (NIMTS #1, 10 June 2009 and Attikon #7, 14 April 2014). The present study followed the STROBE guidelines for observational cross-sectional studies (Supplementary Materials).

### 2.2. Collection of Sociodemographic, Clinical, Lifestyle, and Anthropometric Data

For all participants, interviews were carried out by the same physicians (G.S. and M.D.) to obtain information on demographic, lifestyle (tobacco smoking, diet, alcohol consumption, and physical activity) and reproductive variables (parity, use of hormone replacement therapy and oral contraceptives, age at menarche and menopause, age at first full term pregnancy, and breastfeeding), and medical history. Family history of cancer, including BC was collected for first-degree relatives (parents and siblings) and for second-degree relatives (grandparents, uncles, and aunts). Medical records with cytopathology reports including stage, grade, size, lymph node involvement, and hormone receptor status were recorded. The same physicians performed weight, height, waist circumference (W.C.), and blood pressure (B.P.) measurements under similar conditions at the same time in the morning (8–9.30 am). Moreover, the aforementioned determinations were obtained at the time of diagnosis for cases with BC and BBL, and at the time of the enrolment in the study for control and obese participants. There were no missing data.

BMI was determined based on the formula: body weight (kg)/height^2^ (m^2^). Body fat percentage and fat mass were determined based on the Deurenberg equation [28]. Two BP determinations with the same instrument were performed 5 min apart after 10 min of rest for each participant. BP was calculated by taking into consideration the average of 2 determinations. The Framingham risk score algorithm was used to assess the 10-year general cardiovascular (CVD) risk of subjects [29]. Mets was defined according to the criteria of the National Cholesterol Education Program (NCEP) Adult Treatment Panel (ATP) III [30]. Women presenting three disorders were considered to be low-grade Mets and women presenting more than three disorders (four or five) were considered as high-grade Mets [31,32]. A validated 11-item questionnaire, the MedDietScore, was used to evaluate the adherence to the MedDiet [33,34,35]. This score (from 0 to 55) shows the level of adherence to the MedDiet; a score from 0 to 20 is considered to be low adherence, a score of 21 to 35 is considered to be medium adherence, while a score of 36–55 is considered to be high adherence [33].

### 2.3. Laboratory Determinations

The blood collection was performed under similar conditions early in the morning, after 12 h of fasting, and prior to the initiation of any therapeutic approach for the cases. The peripheral blood samples were centrifuged at 3000 rpm for 5 min in the laboratory. Serum was separated and stored at –80 °C until analysis of adipokine levels, as well as metabolic and tumor biomarkers. This storage temperature guarantees the stability of specimens of a broad range of labile analytes, including adipocytokines, for protracted chronic periods [36]. 

Serum glucose, lipid parameters (total cholesterol, LDL-C, HDL-C, and triglycerides), high-sensitive C-reactive protein (hsCRP), carcinoembryonic antigen (CEA), CA15-3, insulin, interleukin-6 (IL-6), and tumor necrosis factor-α (TNF-α) were determined, as previously described [37,38,39,40,41,42]. The estimate of insulin resistance by homeostasis model assessment score of insulin resistance (HOMA-IR) was calculated using the formula: fasting serum insulin (mU/L) × fasting serum glucose (mmol/L)/22.5. The serum omentin-1 concentrations were measured using a sandwich enzyme immunoassay ELISA kit by Biovendor (#RD191100200R, Brno, Czech Republic). The limit of detection was 0.5 μg/L, the linear range of the assay was reported to be 50–644 μg/L, while the inter-assay and intra-assay coefficients of variations varied within 4.4–4.8% and 3.2–4.1%, respectively.

### 2.4. Statistical Analysis

The statistical analysis was performed using the IBM^®^ SPSS^®^ version 25 for Windows. Categorical variables were presented as numbers and percentages, while continuous variables were presented as a mean ± standard deviation. Data were evaluated through simple cross-tabulations and by using a chi-square test and Fisher’s exact test for categorical variables, a *t*-test for normally distributed variables, and the Mann–Whitney U test for not normally distributed variables. The normality hypothesis was tested by the Kolmogorov–Smirnov test and measures of asymmetry. A one-way analysis of variance (ANOVA) for normally distributed variables or the Kruskal–Wallis test for not normally distributed variables were used for between group comparisons in the cases of more than two groups of continuous variables. The Spearman correlation coefficient (r) and partial correlations were used as the measurements of correlation for continuous or ordinal variables. Statistically significant predictors of circulating omentin-1 (dependent variable) found in univariate linear regression models were introduced in a multiple stepwise linear regression analysis, which was used to identify, in the final model only, significant independent variables affecting circulating omentin-1 (stepwise criteria of entry in the model ≤0.05 and removal from the model ≥0.1) [43]. A multiple unconditional binary logistic regression analysis was used to identify whether omentin-1, expressed also as quartiles, was independently associated with PBC occurrence (dependent variable), adjusting for matching factor and significant clinical and laboratory parameters. Unconditional logistic regression can be used without loss of validity, if the matching factors (age in our study) are accounted for [43]. Skewed continuous variables were logarithmically transformed in the models. A receiver operator characteristic (ROC) curve analysis and calculation of the area under the curve (AUC) were carried out in order to evaluate the ability of serum omentin-1 to correctly distinguish between patients with PBC and controls, patients with BBL, and subjects with obesity. The optimum diagnostic cut-off point for the examined population was chosen to maximize clinical sensitivity and specificity. The level of statistical significance was set at 0.05 for all tests performed. From our earlier clinical study [13], we calculated that we required a total sample size of at least 206 cases and controls to achieve 95% power at the 0.05 level of significance in order to detect a 50 μg/L difference in serum omentin-1.

## 3. Results

### 3.1. Comparisons of Sociodemographic, Lifestyle, Clinical, and Laboratory Variables between Study Groups

**Figure 1:**
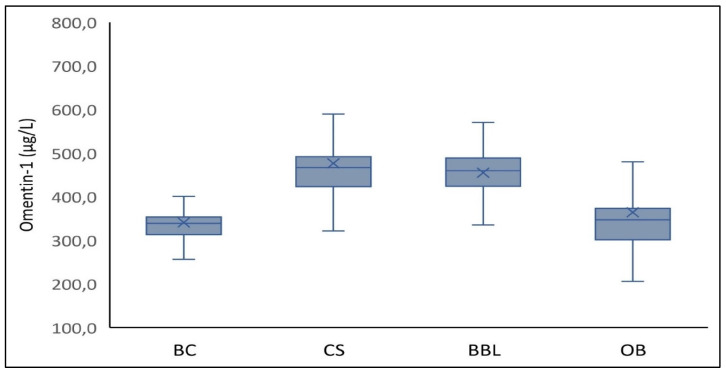
Circulating omentin-1 across study groups: In cases with postmenopausal breast cancer (BC) cases (N = 103), in healthy controls (CS, N = 103), in patients with benign breast lesions (BBL, N = 51), and in postmenopausal participants with obesity (OB, N = 50). Serum omentin-1 levels were significantly lower in BC cases (340.5 ± 109.3 μg/L) than in the control subjects (476.7 ± 156.1 μg/L, *p* < 0.001) and the patients with BBL (455.4 ± 65.9 μg/L, *p* < 0.001), but were similar to the corresponding levels of participants with obesity (363.9 ± 104.4 μg/L, *p* = 0.21).

Table 1 summarizes categorical/ordinal and continuous descriptive features of the study participants. Taking into account variables from matched controls mainly, as well as from patients with BBL and participants with obesity, these data uncovered the majority of established risk factors that characterize cases with PBC such as a positive family history of BC and other cancers; a more frequent use of oral contraceptives or hormones for replacement therapy; a more frequent consumption of alcohol; less physical activity; a more frequent history of Mets (though not statistically significant at 0.05); and less consumption of olive oil, vegetables, and fruits than controls (*p* < 0.05). In comparison to controls, cases with PBC presented an earlier menarche (*p* = 0.04); more years with menstruation (*p* = 0.02); increased weight (*p* = 0.001), BMI (*p* = 0.012), and fat mass (*p* = 0.04); a lower MedDiet score (*p* < 0.001); decreased levels of HDL-C (*p* < 0.001) and adiponectin (*p* = 0.04); higher levels of glucose (*p* = 0.02), total cholesterol (*p* = 0.001), triglycerides (*p* = 0.005), and LDL-C (*p* < 0.001); and a higher CVD risk as expressed by Framingham score (*p* = 0.03). Moreover, cases with PBC tended to have higher WC (*p* = 0.06), mean arterial BP (*p* = 0.06), insulin (*p* = 0.05), and HOMA-IR score (*p* = 0.05) as compared to controls. Overall, tumor markers CEA and CA15-3 were significantly higher in cases than in the controls, patients with BBL, and postmenopausal women with obesity (*p* < 0.001). As portrayed in Figure 1, circulating omentin-1 levels were significantly diminished in cases with PBC (340.5 ± 109.3 μg/L) as compared with the control participants (476.7 ± 156.1 μg/L, *p* < 0.001) and women with BBL (455.4 ± 65.9 μg/L, *p* < 0.001), but were similar to the corresponding levels of participants with obesity (363.9 ± 104.4 μg/L, *p* = 0.21). Interestingly, in every category of participants, individuals with Mets, particularly high-grade Mets, exhibited lower levels of serum omentin-1 (*p* < 0.05). Table 1 also depicts the clinicopathologic variables of cases with PBC including stage, grade, tumor size, lymph node involvement, and hormone receptor status.

### 3.2. Correlations of Omentin-1 with Metabolic, Tumor, Inflammatory, and Clinicopathologic Biomarkers

As depicted in Table 2, in all study participants, circulating omentin-1 displayed negative correlations with consumption of red meat, somatometric variables (BMI, WC, and fat mass), blood pressure indices (SBP, DBP, and mean arterial BP), metabolic and lipid biomarkers (glucose, insulin, HOMA-IR, total cholesterol, triglycerides, and LDL-C), CVD risk, and hsCRP (*p* < 0.05). Omentin-1 presented positive correlations with consumption of olive oil, vegetables and fruits, MedDiet score, and HDL-C (*p* < 0.05). In cases with PBC, serum omentin-1 presented negative associations with tumor biomarkers (CA15-3 and CEA), inflammatory biomarkers (hsCRP, TNF-α, and IL-6), and clinicopathologic features such as stage and the number of infiltrated lymph nodes (*p* < 0.05). In controls, omentin-1 also presented negative correlations with inflammatory biomarkers (hsCRP, TNF-α, and IL-6) and leptin, and a positive correlation with adiponectin (*p* < 0.05). In cases with BBL, omentin-1 also had negative associations with hsCRP and leptin, and a positive correlation with adiponectin (*p* < 0.05). Finally, in obese participants, omentin-1 also presented negative correlations with hsCRP and TNF-α (*p* < 0.05). 

**Figure 2:**
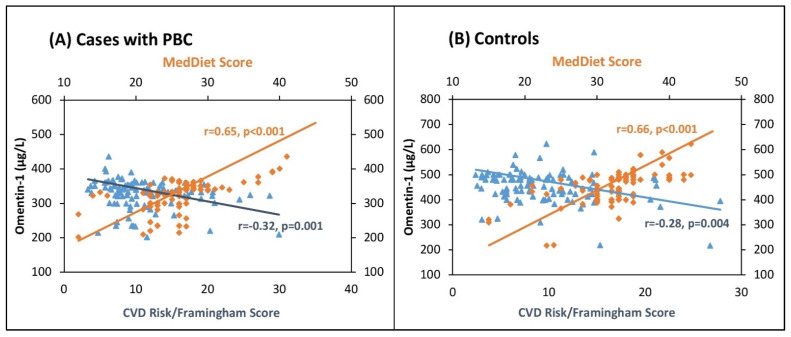
Association of serum omentin-1 with MedDiet Score and cardiovascular risk in 103 cases with postmenopausal breast cancer (**A**) and in 103 control participants (**B**).

Adjusting for BMI, omentin-1 retained the significance of the majority of reported correlations in all study participants except for the correlations between omentin-1 and SBP, DBP, metabolic, and lipid biomarkers in the cases with PBC. Furthermore, adjusting for WC, a proxy of abdominal/visceral adiposity or fat mass, omentin-1 retained the significance of the majority of reported correlations in all study participants except for the correlations between omentin-1 and metabolic and lipid biomarkers in subjects with PBC. Figure 2 graphically depicts the associations of serum omentin-1 with MedDiet score and CVD risk in cases with PBC and in control participants.

### 3.3. Independent Predictors of Circulating Omentin-1

As shown in Table 3, the final model derived from multiple stepwise linear regression shows only the significant predictors of circulating omentin-1 in cases (Model A) and in non-cancer participants (Model B). Adherence to the MedDiet, adiponectin, and mean arterial BP were the independent positive and negative determinants of circulating omentin-1 among all study participants. Moreover, among the PBC cases, hsCRP was a negative predictor, while CA15-3 was a positive predictor. Among non-cancer participants, both IL-6 and fat mass were negative determinants of circulating omentin-1 levels.

### 3.4. Association of Omentin-1 with PBC Occurrence

As shown in Table 4, the multiple logistic regression analysis showed that elevated circulating levels of omentin-1, expressed also as quartiles or SD units, were significantly and inversely associated with PBC occurrence, before and after adjustment for age, BMI, family history of cancer, use of exogenous hormones, alcohol consumption, HOMA-IR score, parity/age at first full term pregnancy, breastfeeding, years with menstruation, adherence tothe MedDiet, adiponectin and leptin concentrations, and serum hsCRP (*p* < 0.001). The same results were obtained for circulating omentin-1 after adjustment for WC (*p* < 0.001), a surrogate measure of abdominal obesity, or fat mass instead of BMI (*p* < 0.001). In a subgroup analysis based on BMI, the inverse significant association between serum omentin-1 levels and PBC occurrence was observed both in participants with BMI less than 30 kg/m^2^ and in participants with BMI equal or above 30 kg/m^2^. 

### 3.5. Association of Circulating Omentin-1 with PBC Clinicopathologic Characteristics

Table 5 shows the circulating omentin-1 levels in cases with PBC by stage, grade, tumor size, lymph node involvement, and hormone receptor status. Serum omentin-1 presented significant associations with stage (*p* = 0.012), lymph node involvement (*p* = 0.04), ER (*p* = 0.03), and ERPR (*p* = 0.02) negative status. In particular, serum omentin-1 tended to decrease in more advanced stage (II and III) but not in stage IV, higher tumor size (*p* = 0.06, though of borderline statistical significance at 0.05), more lymph node involvement, and negative ER or ERPR status. Noteworthy, from Table 2, it was shown that circulating omentin-1 was negatively associated with stage (r = −0.45, *p* < 0.001) and the number of infiltrated lymph nodes (r = −0.48, *p* < 0.001).

### 3.6. Circulating Omentin-1 as a Biomarker in PBC

**Figure 3:**
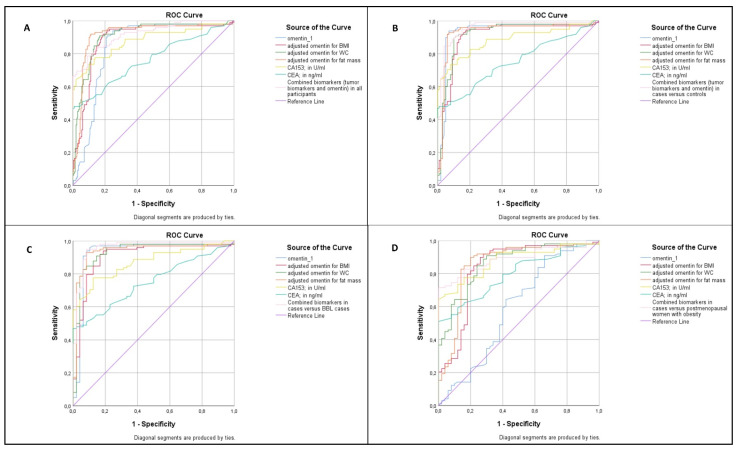
(**A**) Receiver operating characteristic (ROC) analysis of serum omentin-1 (cases with PBC versus controls, patients with BBL, and postmenopausal women with obesity): omentin-1 (AUC (area under the curve) 0.84, 95% CI 0.79–0.89); omentin-1 adjusted for body mass index (BMI) (AUC 0.88, 95% CI 0.84–0.93); omentin-1 adjusted for waist circumference (WC) (AUC 0.90, 95% CI 0.87–0.93); omentin-1 adjusted for fat mass (AUC 0.91, 95% CI 0.87–0.95); CA15-3 (AUC 0.88, 95% CI 0.83–0.93); CEA (AUC 0.76, 95% CI 0.69–0.83); and all biomarkers combined (AUC 0.92, 95% CI 0.89–0.95). (**B**) ROC analysis of serum omentin-1 (cases with PBC versus controls): omentin-1 (AUC 0.93, 95% CI 0.89–0.97); omentin-1 adjusted for BMI (AUC 0.90, 95% CI 0.86–0.95); omentin-1 adjusted for WC (AUC 0.91, 95% CI 0.87–0.96); omentin-1 adjusted for fat mass (AUC 0.93, 95% CI 0.88–0.97); CA15-3 (AUC 0.88, 95% CI 0.83–0.93); CEA (AUC 0.75, 95% CI 0.68–0.82); and all biomarkers combined (AUC 0.95, 95% CI 0.92–0.98). (**C**) ROC analysis of serum omentin-1 (cases with PBC versus patients with BBL): omentin-1 (AUC 0.93, 95% CI 0.87–0.98); omentin-1 adjusted for BMI (AUC 0.90, 95% CI 0.85–0.96); omentin-1 adjusted for WC (AUC 0.93, 95% CI 0.87–0.98); omentin-1 adjusted for fat mass (AUC 0.94, 95% CI 0.89–0.98); CA15-3 (AUC 0.87, 95% CI 0.81–0.93); CEA (AUC 0.75, 95% CI 0.67–0.83); and all biomarkers combined (AUC 0.96, 95% CI 0.92–0.99). (**D**) ROC analysis of serum omentin-1 (cases with PBC versus postmenopausal women with obesity): omentin-1 (AUC 0.59, 95% CI 0.50–0.69); omentin-1 adjusted for BMI (AUC 0.83, 95% CI 0.76–0.91); omentin-1 adjusted for WC (AUC 0.86, 95% CI 0.80–0.92); omentin-1 adjusted for fat mass (AUC 0.85, 95% CI 0.78–0.92); CA15-3 (AUC 0.88, 95% CI 0.82–0.93); CEA (AUC 0.79, 95% CI 0.73–0.87); and all biomarkers combined (AUC 0.89, 95% CI 0.84–0.94).

The ROC analyses of circulating omentin-1, circulating omentin-1 adjusting for BMI or WC or fat mass, tumor biomarkers (CA15-3 and CEA), and all biomarkers combined (omentin-1, CA15-3 and CEA) are given in Figure 3. In the whole study population (Figure 3A), the best cut-off point (based on Youden’s index) for circulating omentin-1 levels was 367 μg/L. Serum omentin-1 levels less than 367 μg/L (cut-off point) yielded a sensitivity and a specificity of 91.3% and 79.4%, respectively, with a good discriminative ability (AUC 0.84, 95% CI 0.79–0.89) for the diagnosis of BC, which is comparable to CA15-3 (*p* = 0.98). The positive and negative predictive values were 69.1% and 94.7%, respectively. Serum omentin-1 was below the optimum cut-off point in 94 patients with PBC (91.3%) versus 6 (5.8%) controls, 3 (5.9%) cases with BBL, and 33 obese postmenopausal women (66%) (χ^2^ = 194, *p* < 0.001). Circulating omentin-1 had a poor discriminative ability (AUC 0.59, 95% CI 0.50–0.69, Figure 3D) as compared with CA15-3 (AUC 0.88, 95% CI 0.82–0.93) for the diagnosis of PBC when comparing cases with PBC to obese postmenopausal women. However, when adjusting for BMI or WC or fat mass, circulating omentin-1 presented better discriminative ability (Figure 3D).

## 4. Discussion

In this cross-sectional study of modest study size, lower circulating omentin-1 levels were independently associated with BC in postmenopausal women, taking into consideration BC risk factors, anthropometric, metabolic, and inflammatory biomarkers. Interestingly, circulating omentin-1 was negatively correlated with cardiometabolic biomarkers, including CVD risk assessed by the Framingham risk score algorithm, and positively associated with MedDiet score. Serum omentin-1 presented a similar diagnostic performance to that of tumor biomarker CA15-3 in BC with a poorer discriminative ability in postmenopausal women with obesity; however, it may reflect advanced disease stage (II and III), increased number of infiltrated lymph nodes, and negative hormonal receptor status. 

The results of our study depicting lower omentin-1 in BC are in agreement with the results of three small case-control studies (N = 30 to 88 premenopausal and postmenopausal case participants) [25,26,27], which provided unadjusted associations due to their small sample size, and the results of a meta-analysis of the two previous studies examining the association of serum omentin-1 with BC occurrence [22,44]. Similar findings for lower omentin-1 levels have also been reported in other malignancies including renal cell cancer [45]. Nevertheless, another study of modest sample size (N = 72 postmenopausal women with treatment-naïve BC) has shown increased serum omentin-1 in treatment-naïve BC patients as compared with healthy controls in unadjusted analyses [24]. 

**Figure 4:**
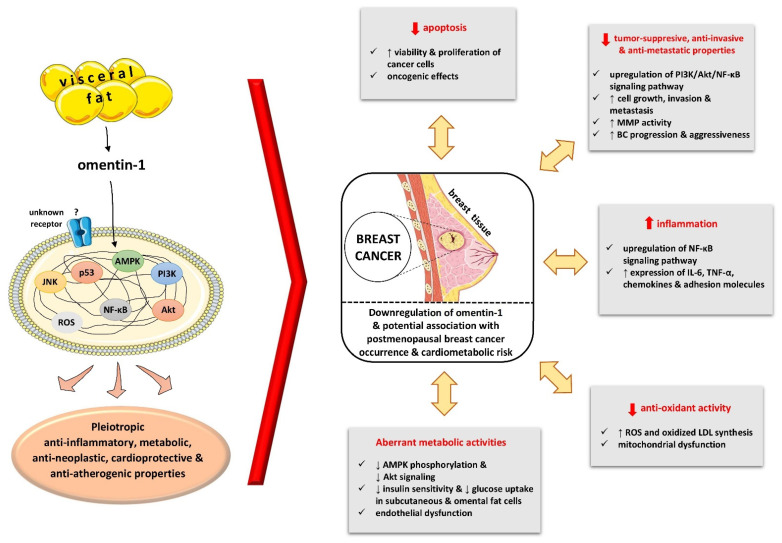
Aberrant expression of omentin-1 in breast cancer and potential implicated mechanisms underlying the association of its lower circulating levels with postmenopausal breast cancer occurrence and cardiometabolic risk. Abbreviations: AMPK, AMP-activated protein kinase; BC, breast cancer; IL-6, interleukin-6; LDL, low-density lipoprotein; MMP, matrix metalloproteinase; NF-κΒ, nuclear factor-κΒ; PI3K, phosphoinositide 3-kinase; ROS, reactive oxygen species; TNF-α, tumor necrosis factor-alpha. (All images are originated from the free medical website http://smart.servier.com/ (accessed on 3 September 2021) by Servier licensed under a Creative Commons Attribution 3.0 Unported License).

A considerable percentage of BC in postmenopausal women is due to obesity due to subclinical chronic inflammation and alterations in hormonal systems, including sex steroid hormones as well as peptide metabolic hormones, such as insulin, bioavailable insulin growth factor (IFG)-1, and adipokines [1,46]. Furthermore, excess visceral adiposity, which is an important source of free fatty acids, proinflammatory cytokines, and adipokines, is independently linked to all criteria constituting Mets as well as BC and CVD risks [31,47,48,49]. Interestingly, omentin-1 is synthesized in the visceral fat, particularly in stromal-vascular cells, and is downregulated in obesity following the same pattern as the anti-inflammatory and pro-apoptotic adipokine adiponectin [50]. Decreased levels of omentin-1 could be a marker of abdominal fat accumulation with an increased risk of BC and CVD [11,18,19,21,51]. Although, the precise pathophysiologic functions of omentin-1 via its unknown cellular receptor have not been elucidated so far, potentially plausible networks underlying the association of lower omentin-1 with PBC risk may include the following (Figure 4):

1. Omentin-1 exhibits pro-apoptotic actions in cancer cells (HepG2 and HuH-7 cells) as observed in hepatocellular carcinoma (HCC) via the activation of the JNK/p53/bcl-2/caspase-3 signaling pathway and the increase and stabilization of p21 and p53 proteins, the latter being a tumor suppressor protein [52]. In vitro studies have also shown that omentin-1 diminished the viability of prostate tumor cells, while knocking down omentin-1 augmented the proliferation of these cells [53].
2. Omentin-1 presents tumor-suppressive, anti-proliferative, anti-invasive, and anti-metastatic potential. In gastric cancer cells, omentin-1 had a negative association with Ki67, an index of cell proliferation, and markers related to invasion and metastasis such as heparanase [54], while it inhibited the progression and metastasis of gastric cancer cells in vivo and in vitro [55]. Similar to our findings in BC, omentin-1 has been found to be inversely associated with tumor stage and lymph node infiltration in gastric cancer patients [54]. Both in vitro and in vivo studies have shown that omentin-1 may attenuate the aggressiveness of gastric cancer cells (invasion and metastasis) via suppression of the phosphoinositide 3-kinase (PI3K)/Akt/Nuclear factor-κΒ (NF-κB) signaling pathway [55]. Interestingly, omentin-1 may influence the growth and aggressiveness of neuroblastoma cells through the upregulation of the N-myc downstream regulated gene 2, which attenuates the expression of vascular endothelial growth factor (VEGF) and matrix metalloproteinase 9 (MMP-9) [56].
3. Omentin-1 has been reported to show anti-inflammatory properties via downregulation of the NF-κΒ signaling pathway, antagonism with the Toll-like receptor-4, and decreased expression of chemokines and cytokines (IL-6, TNF-α, etc.), and adhesion molecules [18]. This is in line with our study findings regarding the inverse relationship of omentin-1 with inflammatory biomarkers such as hsCRP, TNF-α, and IL-6.
4. Omentin-1 exhibits antioxidant potential through the suppression of reactive oxygen species (ROS) and oxidized LDL synthesis, and the protection of mitochondrial function [18,57].
5. Finally, omentin-1 exerts beneficial metabolic properties through the enhancement of insulin’ s actions by activating glucose uptake in subcutaneous and omental fat cells [15], as well as through the prevention of glucose-induced endothelial dysfunction via the stimulation of the AMP-activated protein kinase (AMPK)/peroxisome proliferator-activated receptor δ (PPARδ) pathway [58].

In contrast to cancers presenting with low levels of omentin-1, recent evidence has shown elevated circulating omentin-1 in patients with colorectal, prostate, gastric, and pancreatic cancers [22,54,59,60,61]. This discrepancy may be due, among others, to the different cancer types and locations, the study population, the study design (prospective versus retrospective), the sample size, or the presence of cachexia at an advanced cancer stage, a complex metabolic state characterized by adipose and muscle loss [8,61,62]. Indeed, in our study, higher levels of omentin-1 were observed at stage IV. More and larger studies are needed to explore circulating omentin-1 across BC stages and its significance. 

In addition to BC risk, omentin-1 may function as a biomarker of CVD risk in postmenopausal women [4,14,63]. In agreement with a bulk of previous studies analyzing the associations of omentin-1 with cardiometabolic risk factors, we have found negative associations with anthropometric variables, such as BMI, WC, and fat mass; inflammatory biomarkers, such as hsCRP, TNF-α, and IL-6; metabolic and lipid markers, such as glucose, insulin, HOMA-IR, cholesterol, LDL-C, and triglycerides; and blood pressure parameters, as well as positive associations with cardioprotective and anti-atherosclerotic markers such as HDL-C and adiponectin [19,20,45]. The dyad of adiponectin and omentin-1 are considered to be cardioprotective with beneficial actions in insulin sensitivity and glucose metabolism via the increase of AMPK phosphorylation [63,64,65]. However, it is still unclear whether the regulation of omentin-1 could rely on adiponectin or the opposite effect [20]. In line with previous reported associations, circulating omentin-1 was linked to the presence of Mets and its severity, which is related to an increased occurrence of BC in postmenopausal women [31,66,67].

The findings of our study may present practical and clinical value as omentin-1 could be used as a non-invasive screening and clinical tool of BC in postmenopausal women as well as a preventive and therapeutic target. Low circulating omentin-1 may signal lymph node invasion, negative hormonal receptor status, and increased cardiometabolic risk [25,26,27,63]. More importantly, from a preventive perspective, circulating omentin-1 may increase through weight loss, physical exercise, and the adoption of a healthy diet such as the MedDiet which was found to be a positive predictor of serum omentin-1 in line with another study [68,69]. All three interventions, particularly the adoption of the MedDiet, are beneficial in the prevention of PBC, Mets, obesity, and CVD [7,70,71,72]. The majority of individuals presenting Mets are unaware of their condition and its repercussions [73]. Because of its beneficial metabolic, anti-inflammatory, anti-neoplastic, cardioprotective, and anti-atherogenic properties, omentin-1 could be a promising therapeutic target. Antidiabetic agents, such as metformin, PPAR-γ agonists (pioglitazone), glucagon-like peptide-1 receptor (exenatide), and antilipidemic drugs such as atorvastatin may increase circulating omentin-1 [18]. Interestingly, the administration of omentin-1 delayed the development of atherosclerotic lesions in Apoe(−/−) mice [74].

The strengths of the study include its novelty; the considerable number of clinical and laboratory data; the blind determination of parameters by the laboratory staff; the use of a highly specific ELISA kit for omentin-1 determination; and the enrolment of four groups (newly diagnosed treatment-naïve patients, healthy control participants, cases with BBL, and obese postmenopausal women) of similar age. The limitations of our study include: (1) its cross-sectional nature which cannot demonstrate causality, but could unravel hypotheses to be examined in future large, prospective, and multicentric studies; (2) its modest sample size of cases and controls to enable reliable estimates of joint effects, to identify effect modifiers, and to conduct multivariate subgroup analyses; (3) its small sample size of postmenopausal women with BBL and obesity; (4) its dependence on a single blood sample from study participants, although there are no significant issues regarding reproducibility and reliability in omentin-1 determinations over a chronic time period [75]; (5) the potential of residual confounding by other undetermined variables, which always exist as a possibility in all studies; (6) the non-use of the standard methods of computed tomography (CT) or magnetic resonance imaging (MRI) to quantify abdominal adipose tissue in study participants. Due to ionizing radiation risks, it is not appropriate to determine abdominal fat with CT in many research and clinical studies. While MRI is low risk, it needs specialized resources that are expensive for determining fat distribution alone. Finally, we did not study the expression level of the omentin-1 gene in adipose tissue or cancerous tissue in study participants. 

## 5. Conclusions

In conclusion, in this cross-sectional study of modest sample size, we provided evidence for an independent negative association between circulating omentin-1 and PBC above and beyond known BC risk factors, as well as metabolic and inflammatory parameters. Circulating omentin-1 was negatively associated with cardiometabolic risk factors and positively associated with the MedDiet. Its circulating levels may be modulated by the adoption of the anti-inflammatory and antioxidant MedDiet; however, more, larger studies are needed to confirm this finding. Moreover, circulating omentin-1 as a biomarker presented a similar diagnostic performance to that of tumor biomarker CA15-3 in BC with a poorer discriminative ability in postmenopausal women with obesity. More mechanistic and larger well-designed, multicentric, prospective, and longitudinal studies are required to elucidate the ontological role of omentin-1 in breast tumorigenesis and CVD, its screening and prognostic utility as a non-invasive biomarker, and its therapeutic potential. 

## Figures and Tables

**Table 1:** Descriptive categorical/ordinal and continuous features of postmenopausal breast cancer (PBC) patients (n = 103), healthy control subjects (n = 103), cases with benign breast lesions (BBL) (n = 51), and postmenopausal (PM) women with obesity (n = 50).

Categorical/Ordinal Variables	A	B	C	D	*p*-Value	
	PBC Patients (n = 103) n (%)	Controls (n = 103) n (%)	BBL Cases (n = 51) n (%)	PM Women with Obesity (n = 50) n (%)	All Groups	A vs. Β
Full-term pregnancies (n, %)					**0.03**	0.05
0	24 (23.3)	14 (13.6) ^#^	6 (11.8) ^#^	2 (4) *		
1	13 (12.6)	7 (6.7)	5 (9.8)	2 (4)		
2	54 (52.4)	60 (58.3)	31 (60.8)	36 (72)		
≥3	12 (11.7)	22 (21.4)	9 (17.6)	10 (20)		
Breast-feeding history (n, %)	45 (43.7)	52 (50.5)	23 (45.1)	24 (48) *	**0.04**	0.18
Family history of breast cancer (n, %)	14 (13.6)	4 (3.9) *	1 (2) *	2 (4) ^#^	**0.01**	**0.02**
Family history of cancer (n, %)	37 (35.9)	15 (14.6) *	8 (15.7) *	4 (8) *	**<0.001**	**<0.001**
Previous use of exogenous hormones (oral contraceptives and hormone therapy) (n, %)	16 (15.5)	5 (4.9) *	4 (7.8)	2 (4) *	**0.03**	**0.02**
Physical activity (≥2 h/week) (n, %)	4 (3.9)	10 (9.7) ^#^	6 (11.8) ^#^	0 (0)	**0.03**	0.09
Smoking history (n, %)					0.32	0.26
Non-smoker	63 (61.2)	74 (71.8)	39 (76.5)	30 (60)		
Ex-smoker	8 (7.8)	5 (4.9)	2 (3.9)	2 (4)		
Current smoker	32 (31.1)	24 (23.3)	10 (19.6)	18 (36)		
Passive smoking (n, %)	59 (57.3)	53 (51.5)	23 (45.1)	32 (64)	0.23	0.40
Alcohol consumption (n, %)					**0.04**	0.05
0 glasses/typical day	65 (63.1)	81 (78.6) ^#^	32 (62.7)	34 (68)		
1 glass/typical day	28 (27.2)	16 (15.5)	18 (35.3)	10 (20)		
2 glasses/typical day	8 (7.8)	6 (5.8)	1 (2)	6 (12)		
≥3 glasses/typical day	2 (1.9)	0 (0)	0 (0)	0 (0)		
Education (years) (n, %)					**0.001**	0.07
<6	8 (7.8)	21 (20.4) ^#^	4 (7.8)	6 (12) *		
6	27 (26.2)	31 (30.1)	16 (31.4)	10 (20)		
9	29 (28.2)	22 (21.4)	9 (17.6)	10 (20)		
12	23 (22.3)	15 (14.6)	15 (29.4)	4 (8)		
>12	16 (15.5)	14 (13.6)	7 (13.7)	20 (40)		
Metabolic syndrome (n, %)	36 (35)	24 (23.3) ^#^	13 (25.5)	36 (72) *	**<0.001**	0.07
Grade of Mets (n, % from participants with Mets)					**0.04**	0.09
Low	16 (44.4)	16 (66.7) ^#^	9 (69.2)	13 (36.1)		
High	20 (55.6)	8 (33.3)	4 (30.8)	23 (63.9)		
***Diet variables***						
Adherence to the Mediterranean diet (0–55) (n, %)					0.07	**0.01**
Low (0–20)	5 (4.9)	4 (3.9) *	2 (3.9) *	3 (6)		
Medium (21–35)	91 (88.3)	77 (74.8)	38 (74.5)	41 (82)		
High (36–55)	7 (6.8)	22 (21.4)	11 (21.6)	6 (12)		
Consumption of olive oil in cooking (times per week) (n, %)					**<0.001**	**<0.001**
Never	0 (0)	0 (0) *	0 (0) *	0 (0)		
Rarely	1 (1)	1 (1)	1 (2)	0 (0)		
<1	23 (22.3)	5 (4.9)	6 (11.8)	8 (16)		
1–3	56 (54.4)	36 (35)	21 (41.2)	22 (44)		
3–5	18 (17.5)	41 (39.8)	17 (33.3)	14 (28)		
Daily	5 (4.9)	20 (19.4)	6 (11.8)	6 (12)		
Consumption of red meat (servings per week) (n, %)					0.26	**0.01**
≤1	10 (9.7)	29 (28.2) *	12 (23.5)	9 (18)		
2–3	59 (57.3)	52 (50.5)	28 (54.9)	27 (54)		
4–5	23 (22.3)	16 (15.5)	8 (15.7)	10 (20)		
6–7	10 (9.7)	5 (4.9)	2 (3.9)	3 (6)		
8–10	1 (1)	1 (1)	1 (2)	1 (2)		
>10	0 (0)	0 (0)	0 (0)	0 (0)		
Consumption of vegetables (servings per week) (n, %)					0.41	**0.04**
0	3 (2.9)	1 (1) *	1 (2)	1 (2)		
1–6	81 (78.6)	65 (63.1)	32 (62.7)	36 (72)		
7–12	18 (17.5)	31 (30.1)	16 (31.4)	12 (24)		
13–20	1 (1)	5 (4.9)	2 (3.9)	1 (2)		
21–32	0 (0)	1 (1)	0 (0)	0 (0)		
>33	0 (0)	0 (0)	0 (0)	0 (0)		
Consumption of fruits (servings per week) (n, %)					0.26	**0.04**
0	2 (1.9)	1 (1) *	1 (2)	1 (2)		
1–4	34 (33)	16 (15.5)	9 (17.6)	9 (18)		
5–8	55 (53.4)	65 (63.1)	30 (58.8)	34 (68)		
9–15	11 (10.7)	19 (18.4)	10 (19.6)	6 (12)		
16–21	1 (1)	2 (1.9)	1 (2)	0 (0)		
***Clinicopathologic variables***						
Stage (n, %)		-	-	-	-	-
I	40 (38.8)					
II	31 (30.1)					
III	16 (15.5)					
IV	16 (15.5)					
Grade (n, %)		-	-	-	-	-
I/II	61 (59.2)					
III	42 (40.8)					
Tumor size (n, %)		-	-	-	-	-
<2 cm	45 (43.7)					
≥2 cm	58 (56.3)					
Lymph node involvement (n, %)		-	-	-	-	-
0	23 (22.3)					
1	45 (43.7)					
≥2	35 (34)					
Hormone receptor status (n, %)		-	-	-	-	-
ER+	78 (75.7)					
PR+	57 (55.3)					
ER+ PR+	55 (53.4)					
ER− PR−	24 (23.3)					
**Continuous Variables**	**A**	**B**	**C**	**D**	***p*-Value**	
	**PBC Patients (n = 103)** **Mean (SD)**	**Controls** **(n = 103)** **Mean (SD)**	**BBL Cases** **(n = 51)** **Mean (SD)**	**PM Women with Obesity (n = 50)** **Mean (SD)**	**All Groups**	**A vs. B**
Age, years	61.5 (8.2)	62.3 (8.8)	63.1 (9.6)	61.1 (9.3)	0.52	0.3
***Reproductive Variables***						
Age at menarche (years)	12.2 (1.2)	12.6 (1.2) *	12.61 (0.9)	12.9 (1) *	**0.002**	**0.04**
Age at menopause (years)	49.3 (2.9)	48.5 (3.5) ^#^	48.6 (3.2)	49.6 (2.8)	0.11	0.06
Years with menstruation	37.1 (3.2)	35.9 (3.8) *	35.9 (3.3) *	36.6 (2.9)	0.06	**0.02**
Time since menopause (years)	12.3 (8)	14 (9.5) ^#^	14.3 (9.7)	11.5 (9.7)	0.15	0.09
Age at first full-term pregnancy (years)	28.6 (4.8)	27.3 (5.3) ^#^	27.5 (5.4)	25.7 (4.7) *	**0.02**	0.09
***Somatometric Variables***						
Weight, kg	73.9 (11)	68.1 (13) *	67.7 (11.6) *	88.6 (14.1) *	**<0.001**	**0.001**
Height, m	1.63 (0.06)	1.62 (0.05) ^#^	1.62 (0.05)	1.59 (0.04) *	**<0.001**	0.06
BMI, kg/m^2^	27.7 (4.14)	25.9 (5.4) *	25.7 (4.4) *	35.1 (6) *	**<0.001**	**0.012**
Waist circumference, cm	85.1 (10)	82.3 (10.9) ^#^	84.2 (10.9)	103 (8.6) *	**<0.001**	0.06
Fat mass, %	42 (5.3)	40.2 (6.7) *	39.9 (5.7) *	50.7 (7.7) *	**<0.001**	**0.04**
Fat mass, kg	31.5 (8.5)	28.2 (11.5) *	27.6 (9.3) *	45.9 (15.2) *	**<0.001**	**0.02**
***Blood pressure variables***						
SBP, mmHg	139.7 (17.6)	135.5 (13.5) ^#^	139.2 (19.8)	135.1 (18.2) *	0.19	0.08
DBP, mmHg	82.1 (11.5)	82.3 (9.9)	82.8 (11.1)	83.2 (11.3) *	0.94	0.11
Mean arterial BP ^∂^, mmHg	99 (6.2)	97.2 (7.5) ^#^	97.9 (8.5)	103.9 (8.9) *	**<0.001**	0.06
***MedDiet score***	26.4 (5.3)	32.6 (5.7) *	32.9 (5.2) *	27.4 (7.1)	**<0.001**	**<0.001**
***Metabolic biomarkers***						
Glucose, mmol/L (Ref. range: 4.11–5.88)	5.42 (0.89)	5.14 (0.88) *	5.18 (0.72) ^#^	6.36 (0.92) *	**<0.001**	**0.02**
Insulin, pmol/L (Ref. range: 18.06–172.93)	82.6 (78.5)	65.3 (45.1) ^#^	68.1 (35.4)	109 (54.9) *	**<0.001**	0.05
HOMA-IR score	3.1 (3.7)	2.3 (2.2) ^#^	2.3 (1.5) ^#^	4.6 (3.1) *	**<0.001**	0.05
***Lipid biomarkers and CVD risk***						
Total cholesterol, mmol/L (Ref. range: 3.62–5.69)	5.32 (0.7)	5.01 (0.66) *	5.06 (0.74) *	5.65 (1.03) *	**<0.001**	**0.001**
Triglycerides, mmol/L (Ref. range: <2.26)	1.35 (0.43)	1.18 (0.44) *	1.25 (0.42)	1.77 (0.33) *	**<0.001**	**0.005**
HDL-C, mmol/L (Ref. range ♀: 1.16–1.68)	1.31 (0.15)	1.41 (0.12) *	1.37 (0.14) *	1.25 (0.11) *	**<0.001**	**<0.001**
LDL-C, mmol/L (Ref. range: <4.11)	3.4 (0.65)	3.06 (0.62) *	3.12 (0.71) *	3.6 (0.99)	**<0.001**	**<0.001**
Framingham score, %	10.9 (5.3)	9.3 (5.1) *	10.1 (7.3)	13.1 (7.5) ^#^	**0.003**	**0.03**
***Tumor biomarkers***						
CA 15-3, kU/L (Ref. range: <25)	110.3 (158.1)	17.7 (8.3) *	18.4 (9.3) *	17.5 (9) *	**<0.001**	**<0.001**
CEA, μg/L (Ref. range: <3.8 in NS <5.5 in S)	12.3 (24.6)	1.9 (1.04) *	1.9 (1.05) *	1.6 (0.9) *	**<0.001**	**<0.001**
***Inflammatory biomarkers***						
hsCRP, nmol/L (Ref. range: <47.62)	47.81 (46)	18.95 (17.05) *	19.81 (12.10) *	40 (20.95)	**<0.001**	**<0.001**
TNF-α, ng/L (Ref. range: <8)	23.66 (11.4)	10.35 (4.93) *	10.11 (4.34) *	14.8 (4.6) *	**<0.001**	**<0.001**
IL-6, ng/L (Ref. range: <7)	18.28 (11.39)	8.68 (4.78) *	8.53 (4.94) *	14.1 (5.5) *	**<0.001**	**<0.001**
***Adipokines***						
Leptin, μg/L	28.8 (17.2)	27.8 (17.5)	27.2 (17.8)	43.3 (23.4) *	**0.001**	0.7
Adiponectin, mg/L	16.9 (9.8)	19.8 (10.1) *	18.9 (7.9)	18.5 (8.4)	0.53	**0.04**
Omentin-1, μg/L	340.5 (109.3)	476.7 (156.1) *	455.4 (65.9) *	363.9 (104.4)	**<0.001**	**<0.001**

In bold statistically significant results (*p* < 0.05). ^∂^ Mean arterial BP = (2*DBP + SBP)/3. * *p* < 0.05 between PBC cases and controls, BBL cases or PM women with obesity. ^#^ 0.09 ≤ *p* ≤ 0.05 between PBC cases and controls, BBL cases, or PM women with obesity. Abbreviations: BMI, body mass index; BP, blood pressure; CEA, carcinoembryonic antigen; DBP, diastolic blood pressure; ER, estrogen receptor; HDL-C, high-density lipoprotein cholesterol; HOMA-IR, homeostasis model assessment score of insulin resistance; hsCRP, high-sensitivity C-reactive protein; IL-6, interleukin-6; LDL-C, low-density lipoprotein cholesterol; Mets, metabolic syndrome; NS, non-smokers; PR, progesterone receptor; S, smokers; SBP, systolic blood pressure; SD, standard deviation; TNF-α, tumor necrosis factor-alpha. ♀: female.

**Table 2:** Spearman correlations of study variables with circulating omentin-1 in all participants.

Variables	PBC Patients (n = 103)		Controls (n = 103)		BBL Cases (n = 51)		PM Women with Obesity (n = 50)	
	r	*p*	r	*p*	r	*p*	r	*p*
Smoking history	−0.05	0.59	0.02	0.83	0.05	0.75	0.40	**0.004**
Alcohol consumption	−0.004	0.97	−0.03	0.79	−0.18	0.21	0.10	0.48
Consumption of olive oil	0.52	**<0.001**	0.35	**<0.001**	0.43	**0.002**	0.69	**<0.001**
Consumption of red meat	−0.25	**0.01**	−0.60	**<0.001**	−0.65	**<0.001**	−0.70	**<0.001**
Consumption of vegetables	0.43	**<0.001**	0.61	**<0.001**	0.64	**<0.001**	0.62	**<0.001**
Consumption of fruits	0.49	**<0.001**	0.53	**<0.001**	0.69	**<0.001**	0.74	**<0.001**
***Somatometric Variables***								
BMI	−0.28	**0.004**	−0.47	**<0.001**	−0.48	**<0.001**	−0.44	**0.001**
Waist circumference	−0.29	**0.003**	−0.49	**<0.001**	−0.50	**<0.001**	−0.34	**0.02**
Fat %	−0.24	**0.02**	−0.43	**<0.001**	−0.52	**<0.001**	−0.58	**<0.001**
Fat mass	−0.27	**0.006**	−0.43	**<0.001**	−0.53	**<0.001**	−0.54	**<0.001**
***Blood pressure indices***								
SBP	−0.40	**<0.001**	−0.36	**<0.001**	−0.55	**<0.001**	−0.47	**0.001**
DBP	−0.27	**0.006**	−0.46	**<0.001**	−0.66	**<0.001**	−0.45	**0.001**
Mean arterial BP	−0.38	**<0.001**	−0.45	**<0.001**	−0.61	**<0.001**	−0.49	**<0.001**
***MedDiet score***	0.65	**<0.001**	0.66	**<0.001**	0.70	**<0.001**	0.78	**<0.001**
***Metabolic biomarkers***								
Glucose	−0.26	**0.01**	−0.55	**<0.001**	−0.28	**0.04**	−0.54	**<0.001**
Insulin	−0.21	**0.04**	−0.52	**<0.001**	−0.39	**0.004**	−0,39	**0.005**
HOMA-IR	−0.23	**0.02**	−0.54	**<0.001**	−0.37	**0.008**	−0.47	**0.001**
***Lipid biomarkers and CVD risk***								
Total cholesterol	−0.32	**0.001**	−0.40	**<0.001**	−0.53	**<0.001**	−0.60	**<0.001**
Triglycerides	−0.37	**<0.001**	−0.52	**<0.001**	−0.64	**<0.001**	−0,45	**0.001**
HDL-C	0.35	**<0.001**	0.46	**<0.001**	0.57	**<0.001**	0.33	**0.02**
LDL-C	−0.31	**0.001**	−0.32	**0.001**	−0.47	**0.001**	−0.58	**<0.001**
Framingham score, %	−0.32	**0.001**	−0.28	**0.004**	−0.51	**<0.001**	−0.56	**<0.001**
***Tumor biomarkers***								
CA15-3	−0.39	**<0.001**	0.08	0.41	0.05	0.72	0.12	0.42
CEA	−0.31	**0.002**	−0.05	0.64	−0.03	0.86	0.12	0.42
***Inflammatory biomarkers***								
hsCRP	−0.56	**<0.001**	−0.49	**<0.001**	−0.38	**0.006**	−0.39	**0.005**
TNF-α	−0.51	**<0.001**	−0.50	**<0.001**	−0.23	0.10	−0.39	**0.005**
IL-6	−0.47	**<0.001**	−0.54	**<0.001**	−0.10	0.50	−0.18	0.20
***Adipokines***								
Leptin	−0.001	0.99	−0.27	**0.006**	−0.34	**0.02**	−0.22	0.13
Adiponectin	−0.02	0.82	0.21	**0.03**	0.45	**0.001**	0.26	0.07
***Clinicopathologic features***								
Stage	−0.45	**<0.001**	-	-	-	-	-	-
Number of infiltrated lymph nodes	−0.48	**<0.001**	-	-	-	-	-	-

In bold statistically significant results (*p* < 0.05). Abbreviations: BMI, body mass index; BP, blood pressure; CEA, carcinoembryonic antigen; DBP, diastolic blood pressure; HDL-C, high-density lipoprotein cholesterol; HOMA-IR, homeostasis model assessment score of insulin resistance; hsCRP, high-sensitivity C-reactive protein; IL-6, interleukin-6; LDL-C, low-density lipoprotein cholesterol; SBP, systolic blood pressure; TNF-α, tumor necrosis factor-alpha.

**Table 3:** Multiple linear regression analysis (stepwise method) depicting independent predictors of circulating omentin-1 levels (as dependent variable) in 103 patients with postmenopausal breast cancer (Model A) and in 103 age-matched controls, 51 cases with benign breast lesions, and 50 postmenopausal women with obesity (Model B); regression coefficients (b), standard error of b (SE_b_) and t statistic with corresponding *p*-value.

Model A in Patients with Postmenopausal Breast Cancer (n = 103)					Model B in Controls, Cases with BBL, and Postmenopausal Women with Obesity (n = 204)				
Independent Variables	b	SE_b_	t Statistic	*p*-Value	Independent Variables	b	SE_b_	t Statistic	*p*-Value
Adherence to the Mediterranean diet	98.10	31.43	3.12	**0.002**	Adherence to the Mediterranean diet	96.44	14.60	6.61	**<0.001**
Adiponectin (mg/L)	2.56	1.01	2.53	**0.013**	Adiponectin (mg/L)	2.12	0.73	2.89	**0.004**
Mean arterial BP (mmHg)	−3.85	1.79	−2.15	**0.03**	Mean arterial BP (mmHg)	−2.39	0.97	−2.47	**0.014**
hsCRP (nmol/L)	−11.58	2.71	−4.27	**<0.001**	IL-6 (ng/L)	−3.36	1.36	−2.47	**0.014**
CA 15-3 (kU/L)	0.28	0.08	3.42	**0.001**	Fat mass (%)	−3.44	1.08	−3.19	**0.002**

In bold statistically significant results (*p* < 0.05). Abbreviations: BP, blood Pressure; hsCRP, high-sensitivity C-reactive protein; IL-6, interleukin-6.

**Table 4:** Odd ratios (OR) and 95% confidence intervals (CI) for postmenopausal breast cancer in relation to serum omentin-1 by control-defined quartiles, SD and continuously.

Serum Omentin-1 (μg/L)	Category or Increment	Crude OR (95% CI)	*p*-Value	Adjusted OR ^a^ (95% CI)	*p*-Value
**As compared with controls**					
Omentin-1 in quartiles	1 quartile more	0.08 (0.04–0.16)	**<0.001**	0.006 (0.001–0.04)	**<0.001**
Omentin-1 (continuous variable)	1 μg/L of omentin-1more	0.98 (0.97–0.98)	**<0.001**	0.98 (0.97–0.99)	**<0.001**
Omentin-1 SD	1 SD unit of omentin-1 more	0.03 (0.01–0.07)	**<0.001**	0.01 (0.003–0.05)	**<0.001**
**As compared with controls, BBL cases, and PM participants with obesity**					
Omentin-1 in quartiles	1 quartile more	0.23 (0.16–0.32)	**<0.001**	0.04 (0.02–0.09)	**<0.001**
Omentin-1 (continuous variable)	1 μg/L of omentin-1 more	0.99 (0.98–0.99)	**<0.001**	0.98 (0.97–0.98)	**<0.001**
Omentin-1 in SD	1 SD unit of omentin-1 more	0.09 (0.05–0.18)	**<0.001**	0.01 (0.003–0.04)	**<0.001**

Abbreviations: CI, confidence interval; OR, odds ratio; PM, postmenopausal; SD, standard deviation. ^a^ OR adjusted for age, body mass index, family history of cancer, use of exogenous hormones, alcohol consumption, HOMA-IR score, parity, age at first full term pregnancy, breastfeeding, years with menstruation, adherence to the MedDiet, adipokines, and serum hsCRP.

**Table 5:** Serum omentin-1 (μg/L) and its association with clinicopathologic features in 103 cases suffering from postmenopausal breast cancer.

Clinicopathologic Features	Serum Omentin-1, μg/L (Mean ± SD)	*p*-Value
**Stage**		**0.012**
I	369.1 ± 123.9	
II	331.3 ± 17.3	
III	267.1 ± 41.7	
IV	358.4 ± 169.4	
**Grade**		0.10
I/II	355.1 ± 102.4	
III	319.3 ± 116.7	
**Tumor size**		0.06
<2 cm	362.7 ± 118.4	
≥2 cm	323.2 ± 99.3	
**Lymph node involvement**		**0.04**
0	388.9 ± 160.5	
1	335 ± 21.3	
≥2	315.6 ± 127.8	
**ER status**		**0.03**
Positive	349.3 ± 122.2	
Negative	312.9 ± 42.7	
**PR status**		0.12
Positive	354.4 ± 141.2	
Negative	323.2 ± 41.7	
**ERPR status**		0.14
Both positive	354.6 ± 143.7	
Other types	324.2 ± 41.3	
**ERPR status**		**0.02**
Both negative	311.2 ± 42.5	
Other types	349.3 ± 121.5	

In bold statistically significant results (*p* < 0.05). Abbreviations: ER, estrogen receptor; PR, progesterone receptor; SD, standard deviation.

## Data Availability

Data to support the findings of this study are available upon reasonable request.

## Supplementary Materials

The following are available online at https://www.mdpi.com/article/10.3390/biom11111609/s1, Table S1: STROBE checklist for observational studies.
